# Evaluation of aqueous humor and serum clusterin levels in patients with glaucoma

**DOI:** 10.1186/s12886-020-01781-7

**Published:** 2021-01-09

**Authors:** Fuat Yavrum, Ufuk Elgin, Zeynep Adiyaman Kocer, Vildan Fidanci, Emine Sen

**Affiliations:** 1 Patnos State Hospital, 4500 Ağrı, Turkey; 2 University of Health Sciences, Ulucanlar Eye Research Hospital, Ankara, Turkey; 3 University of Health Sciences, Ankara Research and Training Hospital, Ankara, Turkey

**Keywords:** Clusterin, Pseudoexfoliation syndrome, Pseudoexfoliation glaucoma, Primary open-angle glaucoma

## Abstract

**Background:**

To compare the aqueous humor (AH) and the serum clusterin levels of patients with pseudoexfoliation syndrome (PEX), pseudoexfoliation glaucoma (PEXG), and primary open-angle glaucoma (POAG) with each other and with an age- and sex-matched control group.

**Methods:**

This prospective, cross-sectionalstudy evaluated 92 eyes from 92 adult cases of uncomplicated phacoemulsification and posterior chamber intraocular lens (IOL) implantation. The cases were divided into PEX, PEXG, POAG, and control groups. Serum samples were taken from the antecubital vein just before the surgery, and the AH samples were aspirated at the beginning of the surgery. Kruskal-Wallis H, One-way ANOVA, Mann-Whitney U with Bonferroni correction and Chi-Square tests were used for statistical analysis.

**Results:**

The serum clusterin levels were the highest in the PEXG group, but no statistically significant differences were observed between the groups (*p*=0.633). The mean AH clusterin levels were 286.79±29.64 μg/mL in the PEXG group, 263.92±31.70 μg/mL in the PEX group, 272.59±49.71 μg/mL in the POAG group, and 193.50±62.38 μg/mL in the control group (*p*< 0.001). This came out to be 1.48 times increase for the PEXG group, 1.36 for the PEX group, and 1.41 for the POAG group when compared with the control subjects.

**Conclusions:**

A higher level of clusterin in the anterior chamber was found to be associated with PEX and PEXG. In addition, a high level of anterior chamber clusterin in POAG, which is a new finding, showed that this molecule might be important not only in pseudoexfoliation, but also other types of glaucoma like POAG.

## Background

Glaucoma is a progressive, multifactorial optic neuropathy that is characterized by both visual acuity and visual-field loss. Worldwide, it is the second most common cause of blindness after cataract [1]. Primary open-angle glaucoma (POAG) is the most common form of this disease and tends to develop without other known systemic or ocular conditions [2].

Pseudoexfoliation syndrome (PEX) is a systemic, age-related disorder of the extracellular matrix and is the most common cause of secondary open-angle glaucoma [3, 4]. Pseudoexfoliation material (PEXM) progressively accumulates in the trabecular meshwork and increases the outflow resistance of the aqueous humor (AH), leading to an increase in intraocular pressure (IOP) [4]. Pseudoexfoliation glaucoma (PEXG) is a secondary open-angle glaucoma that is characterized by its rapid progression, resistance to medical treatment, and a worse prognosis compared to POAG [5].

Clusterin is a 70–80 kDa lipoprotein which is produced in almost all tissues and is present in all body fluids [6, 7]. In the eye, it has been observed in the cornea, lens, ciliary body, retina, AH, and the vitreous [8]. It is a multifunctional protein that has roles in a wide range of physiological and pathological processes such as lipid transport, apoptosis, cell-cell and cell-matrix interactions, and the folding of denatured proteins as a result of stress [9]. Clusterin acts as a chaperone protein that converts precursor or denatured proteins into their active forms [10], and its expression has been shown to increase in cases of cellular damage such as heat shock, UV radiation, and oxidative stress [6, 11]. In recent studies, the clusterin molecule has been demonstrated in both PEX fibrils on the lens and PEXM in the trabecular network [12–14]. In addition, Doudevski et al. [15] investigated both serum and AH clusterin levels and showed higher levels of AH clusterin in PEX cases compared to control subjects. In contrast, a similar study by Zenkel et al. [16] reported lower levels of AH clusterin in the PEX group compared to other glaucoma types and to controls.

As clusterin has been demonstrated to be related to the pathogenesis of PEX and PEXG, we hypothesized that there might be differences between clusterin levels in glaucomatous and nonglaucomatous eyes. We therefore compared AH and serum clusterin levels in patients with PEX, PEXG, and POAG to each other and to age- and gender-matched controls to further explore their pathophysiologies.

## Methods

This prospective, cross-sectional study was conducted in the Glaucoma Department of the University of Health Sciences, Ulucanlar Eye Research Hospital, between March, 2018 and January, 2019. The study was conducted in accordance with the ethical standards of the Declaration of Helsinki, and its protocol was approved by the ethics committee of the Numune Training and Research Hospital. Written informed consent was obtained from all patients for the analyzed cases before the study began.

For each subject, a detailed ophthalmological examination was conducted, including best corrected visual acuity (BCVA) with Snellen charts and logMAR (logarithm of minimum angle of resolution) values, biomicroscopic and fundus examinations, IOP and central corneal thickness (CCT) measurements with Goldmann applanation tonometer and ultrasonic pachymeter, at least three reliable consecutive visual field examinations with Humphrey automated perimeter, and an optic disk and retinal nerve-fiber layer investigation using spectral-domain optic coherence tomography (OCT) (Spectralis, Heidelberg Engineering, Germany).

All patient participants had senile cataracts and were hospitalized for cataract surgery. The inclusion criteria for all groups were: age older than 50 years; and BCVA between hand motion and 20/40 vision. The inclusion criteria for the POAG group were: IOP under control by antiglaucomatous agents; Grade 3–4 open-angle according to the Shaffer angle grading system; optic nerve head changes (e.g., cup to disk ratio ≥0.3, localized neuro-retinal rim defects, peripapillary choroidal atrophy, and splitter hemorrhage); presence of glaucomatous visual field defects (e.g., abnormal glaucoma hemifield test, nasal step, seidel, or arcuate scotoma); and the presence of a glaucomatous nerve fiber layer and optic disk in the OCT findings. The inclusion criteria for the PEXG group were: the presence of PEXM on the surface of the lens and/or pupillary margin; and all POAG inclusion criteria. The inclusion criteria for the PEX group were: the presence of PEXM on the surface of the lens and/or pupillary margin; IOP values< 21 mmHg without any antiglaucomatous agents; and no glaucomatous findings during the clinical, visual field, and OCT examinations. Glaucoma was determined to be under control for all of the glaucoma cases. Patients with early and moderate visual field defects according to Hodapp-Parrish-Anderson staging system (mean deviation (MD) less than − 12 dB) were included [17]. The control subjects did not have any systemic or ocular problems other than senile cataracts.

The exclusion criteria were: patients with glaucoma types other than POAG and PEXG; any history of any ocular trauma, surgery, uveitis, macular diseases (e.g., age-related macular degeneration), or optic nerve diseases (e.g., ischemic optic neuropathy); any history of neurologic, psychiatric, neurodegenerative or neurosurgical systemic problems. Also, eyes from patients with severe visual field defects (MD greater than − 12 dB) were excluded.

### Sample examination

All serum samples were taken from the antecubital vein just before surgery and then transferred to a biochemistry tube containing a separator gel. After the blood samples were sent to the laboratory, they were centrifuged (Eppendorf 5810, Hamburg, Germany) at 3000 rpm for 10 min, and the obtained serum stored at − 80 °C (SANYO MDF U6186, Osaka, Japan) until analysis.

The AH samples were obtained using a 1 ml syringe immediately after entering the anterior chamber, and all samples collected during surgery were obtained before any cataract-surgery related medications were applied. The samples were stored at − 80 °C until analysis. The concentrations of clusterin were assessed using a Human Clusterin PicoKine ELISA Kit (MyBioSource, Catalog No. MBS176539, LOT 5601235403, CA, USA) according to the manufacturer’s instructions. The author performing the clusterin analyses (VF) was blinded to patient details. Anterior chamber concentration units were μg/ml, those for serum samples were ng/ml.

### Statistical analysis

The data obtained from the study were analyzed using the Statistical Package for Social Sciences (SPSS) version 20.0 for Windows (SPSS, Inc., Chicago, IL, USA). The chi-square test was used to analyze categorical values. Continuous group variables were evaluated using a one-way analysis of variance (ANOVA) or Kruskal-Wallis H tests, depending on whether the variables were normally distributed. Similarly, either Independent Samples *T*-test or Mann-Whitney U tests were used for pairwise comparisons between groups according to the variable characteristics. Spearman’s test was used for the correlation analyses due to the non-normal distribution of the variables. A *p* value less than 0.05 was considered significant.

## Results

Ninety-two eyes from 92 cases were included in the current study. Twenty-four of these cases were in the PEXG group, 24 were in the PEX group, 22 were in the POAG group, and 22 were in the control group. A total of eight patients were excluded from the study: three from the PEXG group; two from the POAG group; two from the PEX group; and one from the control group due to insufficient anterior-chamber sample collection during surgery.

The patient demographic characteristics for the groups are summarized in Table 1. There were no significant differences in age, gender, BCVA, and IOP between the groups (*p*=0.674, *p*=0.756, *p*=0.971, and *p*=0.119, respectively).

**Table 1:** Patient demographic and clinical features of the PEX, PEXG, POAG, and control groups

	PEXG	PEX	POAG	CONTROL	*P*
Age (years), mean ± SD (Range)	69.88 ± 9.39 (51–85)	69.83 ± 7.20 (58–84)	68.32 ± 9.01 (51–83)	67.32 ± 7.35 (52–82)	0.674^*^
Female/Male (n/n)	10/14	12/12	9/13	12/10	0.756^**^
BCVA (logMAR), mean ± SD	0.80±0.81	0.76±0.67	1.06±0.84	0.93±0.76	0.971^***^
IOP (mmHg), mean ± SD	17.71±3.75	16.25±2.58	18.59±4.30	16.00±1.93	0.119^***^

*PEXG* Pseudoexfoliation glaucoma, *PEX* Pseudoexfoliation syndrome, *POAG* Primary open angle glaucoma, *SD* Standard deviation

^*^One-way analysis of variance (ANOVA)

^**^Chi-square test

^***^Kruskal-Wallis H test

For the PEXG and POAG groups, the mean values for the number of antiglaucomatous agents were 2.08 and 2.14, respectively. The use of prostaglandin analogue monotherapy (PGA) was 41.7% (*n*=10) in the PEXG group, and 63.6% (*n*=14) in the POAG group. There was no statistically significant difference in the number of the antiglaucomatous agents between the groups (*p*=0.136).

The mean serum clusterin levels were 672.67±323.18 ng/ml (range: 255–1290 ng/ml) in the PEXG group, 625.08±261.14 ng/ml (range: 148–1137 ng/ml) in the PEX group, 632.77±385.30 ng/ml (range: 104–1382 ng/ml) in the POAG group, and 553.05±281.65 ng/ml (range: 129–1074 ng/ml) in the control group. Serum clusterin levels were highest in the PEXG group, but no statistically significant differences were observed between the groups (*p*=0.633) (Table 2).

**Table 2:** Mean values for serum and aqueous humor clusterin levels among the groups

Clusterin	PEXG (***n***=24)	PEX (n=24)	POAG (***n***=22)	Control (***n***=22)	P*
**Serum (ng/mL)** **(mean±SD)**	672.67±323.18	625.08 ± 261.14	632.77 ±385.30	553.05 ±281.65	0.633
**AH (μg/mL)** **(mean±SD)**	286.79 ±29.64	263.92 ±31.70	272.59 ±49.71	193.50 ±62.38	**< 0.001**

*PEXG* Pseudoexfoliation glaucoma, *PEX* Pseudoexfoliation syndrome, *POAG* Primary open angle glaucoma, *SD* Standard deviation

*Kruskal-Wallis H test

*Bold values show statistical significance.*

The mean AH clusterin levels were 286.79±29.64 μg/ml (range: 209–329 μg/ml) in the PEXG group, 263.92±31.70 μg/ml (range: 210–319 μg/ml) in the PEX group, 272.59±49.71 μg/ml (range: 168–356 μg/ml) in the POAG group, and 193.50±62.38 μg/ml (range: 102–310 μg/ml) in the control group. The mean AH clusterin levels showed a high level of statistical significance among the groups (*p*< 0.001) (Table 2).

There were positive correlations between serum and AH clusterin levels among all patients, but these correlations were not statistically significant (*p*=0.394, r=0.09).

**Fig. 1:**
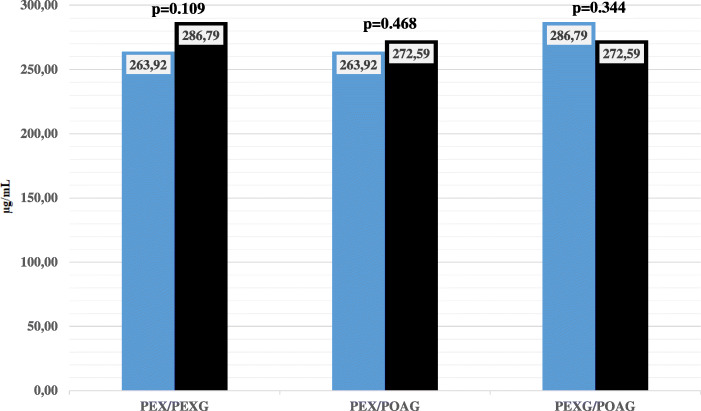
Pair wise comparison between POAG, PEXG and PEX groups in terms of AH clusterin level (Mann-Whitney U was used for analyses)

**Fig. 2:**
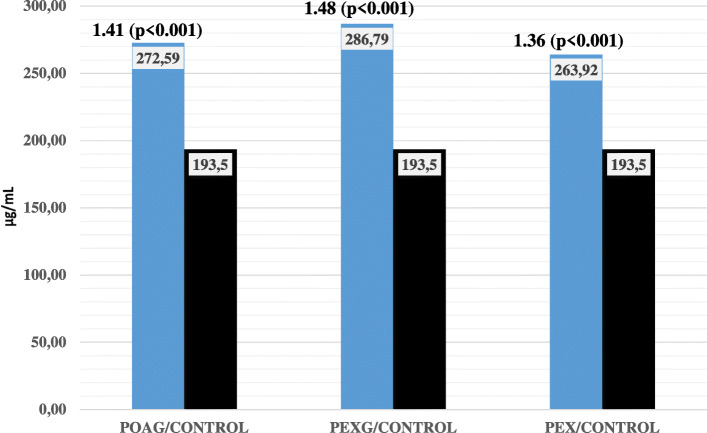
Pair wise comparison between POAG, PEXG, and PEX groups with control group in terms of AH clusterin level (Mann-Whitney U was used for analyses)

The pairwise comparisons for the PEX, PEXG, and POAG groups showed no significant differences (Fig. 1). However, comparisons of clusterin AH values between groups were significantly different, with clusterin levels 1.48-fold higher in the PEXG group, 1.36-fold higher in the PEX group, and 1.41-fold higher in the POAG group compared to control subject values (*p*< 0.001, *p*< 0.001, and *p*< 0.001, respectively). (Fig. 2).

## Discussion

Here, we compared both the AH and serum levels of clusterin in PEX, PEXG, POAG, and control subjects and found significantly higher levels of AH clusterin in PEXG, POAG, and PEX cases compared to control-group cases. Our primary aim was to better understand the pathophysiology of clusterin because of the contradictory findings reported [14–16].

The pathogenesis of glaucoma, which is among the most common causes of blindness worldwide, is still unclear. Although many factors, including vascular, immunological, genetic, and mechanical and oxidative stress are known to be involved in its pathogenesis, the relative importance of inflammation has also increased recently [1, 2]. Moreover, the pathogeneses for both PEX and PEXG are known to be multifactorial [3, 4].

Yildirim et al. [18] examined the relationship between PEX and inflammatory cytokines and found that serum IL-6 levels were significantly higher in a PEX group when compared to controls. As a result, subclinical inflammation and blood-aqueous barrier disorder were found to be correlated with high IL-6 levels in PEX patients. Kondkar et al. [19] investigated the plasma levels of another inflammatory marker in PEX cases, tumor necrosis factor alpha (TNF-α), and concluded that high TNF-α levels may be a marker for the progression of PEX to PEXG, especially with the highest plasma levels. Similarly, Eraslan et al. [20] compared the levels of ghrelin (an amino-acid hormone), acylated ghrelin, and the ratio of acylated ghrelin to ghrelin in PEX, PEXG, POAG, and control patients. They reported high acylated ghrelin/ghrelin ratios in PEXG cases, and concluded that acylated ghrelin might play a role in the transformation of PEXG by negatively regulating prostaglandin and nitric oxide release [20].

The clusterin glycoprotein consists of both alpha and beta chains that bind to each other through disulfide bonds; it is expressed in many tissues in the body, and is located in extracellular regions and in body fluids [6–11]. It has important roles in the clustering of cells, complement inhibition, lipid transport, inflammation, and apoptosis [20]. The relationship between clusterin and both PEX and PEXG has been shown in numerous studies [14–16]. Padhy et al. [14] investigated whether clusterin might be a risk factor for PEX. They compared lens capsule and AH clusterin protein levels in PEX, PEXG, and control groups using immunohistochemistry and Western blot techniques. They found higher clusterin protein levels in the PEXG group compared to the control group and concluded that increased clusterin in the AH and lens capsule might explain the progression from PEX to PEXG, by specifically causing more protein deposition [14]. Doudevski et al. [15] examined PEXM lens deposits using high-resolution microscopy and confocal immunofluorescence methods to measure clusterin, vitronectin, and complement 3a and 5b-9 levels. They found that clusterin and vitronectin levels were 1.7-fold higher in the PEXG group when compared to the POAG group.

Here, we found AH clusterin levels to be significantly higher in the PEXG group compared to the control group. In addition, mean serum clusterin levels were higher in the POAG and PEXG groups compared to PEX and control-group levels, but these differences were not statistically significant. Indeed, the present results are similar to those of both Padhy et al. [14] and Doudevski et al. [15]. Zenkel et al. [16] investigated the AH clusterin expression in cases of PEX, PEXG, POAG, primary angle-closure glaucoma (PACG), and controls using real-time PCR, western blot, and immunohistochemical methods. They reported lower AH clusterin levels in PEX eyes compared to levels in POAG, PACG, and control-group eyes. However, no significant clusterin differences were found between PEX and PEXG eyes. As a result, they concluded that a clusterin deficiency in the anterior segment may have caused a stress-related, pathological accumulation of extracellular matrix [16]. These controversial results could be due to differences in sample sizes, patient ages, and clusterin evaluation methods. According to Padhy et al. [14], Doudevski et al. [15], and our study, the elevated AH clusterin levels in PEXG cases can be explained by clusterin leakage from serum to the AH as a result of an impaired blood-aqueous barrier due to glaucomatous stress.

In a recent study by Can Demirdogen et al. [21], AH and tear-fluid levels of clusterin were compared between PEX, PEXG, and control subjects. They found significantly higher levels of AH clusterin in the PEXG group compared to PEX and control subjects, no significant differences were found between groups for tear-fluid levels. They concluded that AH clusterin could distinguish patients with PEXG and from those with PEX, and that tear clusterin was not likely to be a predictive biomarker for PEXG or for PEX [21].

In contrast to the above study [21], we examined clusterin serum levels in addition to AH levels and found no significant serum-level differences between groups. This result is consistent with the findings of Zenkel et al. [16], emphasizing that clusterin only has a local effect on PEX eyes. In our PEXG group, AH clusterin levels were highest, followed by levels in the POAG, PEX, and control groups, respectively. These increases in AH clusterin levels for cases of PEXG and POAG were also reported by Zenkel et al. [16], and explained as a deterioration of the blood-aqueous barrier due to glaucoma and leakage of systemic clusterin into the AH. On the other hand, Doudevski et al. [15] argued that this increase could not be explained by the breakdown of the blood-aqueous barrier alone, and that local synthesis might therefore play an important role. In the present study, the higher levels of AH clusterin in PEXG cases compared to POAG cases may be explained by the more aggressive nature of PEXG compared to POAG in the deterioration of the blood-aqueous barrier. Moreover, blood-aqueous barrier disorders in glaucoma patients may also result from the use of prostaglandin agents in most cases. Indeed, as reported by Nakamura-Shibasaki et al. [22], prostaglandins are known to disrupt the blood-aqueous barrier. In their animal study, prostaglandin E2 administration disrupted the blood-aqueous barrier and increased the amount of flare in the anterior chamber.

We also found higher clusterin levels in the anterior chambers of POAG cases compared to control-group levels. This might also be due to leakage of systemic clusterin into the AH as a result of deterioration of the blood-aqueous barrier due to glaucomatous stress and prostaglandin use. As clusterin is a multifunctional protein, its expression has also been reported to increase markedly in response to many other types of cellular stress, including oxidative stress, an important factor involved in glaucoma pathogenesis [23]. Therefore, the local production of clusterin due to oxidative stress may also have influenced our results.

Here, the lower AH clusterin levels in the PEX group, compared to those of the PEXG and POAG groups, are consistent with the findings of Zenkel et al. [16]. However, we also found higher AH clusterin levels in the PEX group compared to the control-group levels. This difference can be explained by the presence of subclinical inflammation and deterioration of the blood-aqueous barrier in the PEX group.

There are limitations for this study: the group sizes were relatively small; no primary angle-closure glaucoma group was included; and we only investigated the relationship between glaucoma types and clusterin, without considering any relationships between disease severity, IOP, and clusterin levels.

## Conclusions

Both PEX and PEXG cases were found to be associated with higher clusterin levels in the anterior chamber. In addition, POAG cases demonstrated higher levels of anterior chamber clusterin, which to the best of our knowledge, has not been previously reported. Based on these findings, clusterin may be important not only in PEX, but also in all glaucoma types. Further studies, with a higher number of cases and different types of glaucoma, are warranted to clarify the pathogenesis of this disease.

## Data Availability

The datasets used and/or analyzed during the current study are available from the corresponding author on reasonable request.

## Abbreviations

AH: Aqueous humor
BCVA: Best corrected visual acuities
CCT: Central corneal thickness
IOL: Intraocular lens
IOP: Intraocular pressure
OCT: Optic coherence tomography
PACG: Primary angle-closure glaucoma
PEX: Pseudoexfoliation syndrome
PEXG: Pseudoexfoliation glaucoma
PEXM: Pseudoexfoliation material
POAG: Primary open-angle glaucoma
TNF-α: Tumor necrosis factor alpha
